# Kinetic characterization of *Vibrio cholerae* ApbE: Substrate specificity and regulatory mechanisms

**DOI:** 10.1371/journal.pone.0186805

**Published:** 2017-10-24

**Authors:** Xuan Fang, Pingdong Liang, Daniel Alexander Raba, Mónica Rosas-Lemus, Srinivas Chakravarthy, Karina Tuz, Oscar Juárez

**Affiliations:** 1 Department of Biological Sciences, Illinois Institute of Technology, Chicago, Illinois, United States of America; 2 Biophysics Collaborative Access Team, Advanced Photon Source, Argonne National Laboratory, Lemont, Illinois, United States of America; Russian Academy of Medical Sciences, RUSSIAN FEDERATION

## Abstract

ApbE is a member of a novel family of flavin transferases that incorporates flavin mononucleotide (FMN) to subunits of diverse respiratory complexes, which fulfill important homeostatic functions. In this work a detailed characterization of *Vibrio cholerae* ApbE physiologic activity, substrate specificity and pH dependency was carried out. The data obtained show novel characteristics of the regulation and function of this family. For instance, our experiments indicate that divalent cations are essential for ApbE function, and that the selectivity depends largely on size and the coordination sphere of the cation. Our data also show that ApbE regulation by pH, ADP and potassium is an important mechanism that enhances the adaptation, survival and colonization of *V*. *cholerae* in the small intestine. Moreover, studies of the pH-dependency of the activity show that the reaction is favored under alkaline conditions, with a pKa of 8.4. These studies, together with sequence and structure analysis allowed us to identify His257, which is absolutely conserved in the family, as a candidate for the residue whose deprotonation controls the activity. Remarkably, the mutant H257G abolished the flavin transfer activity, strongly indicating that this residue plays an important role in the catalytic mechanism of ApbE.

## Introduction

Most enzymes bind flavin cofactors through non-covalent interactions, including hydrogen bonding [1–3] and aromatic-aromatic (Pi-Pi) interactions [3,4]. However, in approximately 10% of flavoproteins the flavin adenine dinucleotide (FAD) or flavin mononucleotide (FMN) molecules are covalently-linked to the protein, mainly through bonds of the isoalloxazine ring [5] with histidine [6–8], cysteine [9,10], tyrosine [11], or with histidine and cysteine residues together [12,13]. Recent reports indicate that some bacterial respiratory enzymes contain covalently-attached FMN cofactors, linked via a unique phosphoester bond between the phosphate group of FMN and the hydroxyl group of the last threonine residue in the semiconserved sequence: S(T)GAT [14–19]. These enzymes include the Na^+^-pumping NADH dehydrogenase (Na^+^-NQR) [15–17], the related membrane-bound ferredoxin reductase RNF [18], urocanate reductase [17,20] and NosR, putatively part of a respiratory pathway involved in denitrification reactions [17,19,21]. Among these respiratory enzymes Na^+^-NQR stands out as essential in the physiology of many types of pathogenic bacteria, including *V*. *cholerae*, *Chlamydia trachomatis*, *Klebsiella pneumoniae*, etc. [22–26]. This complex transfers electrons into the bacterial respiratory chain [22], and creates a sodium gradient [27] that fulfills essential physiologic functions, such as flagella rotation, ATP synthesis, nutrient transport, and virulence factor secretion [23,25,28,29]. The sodium gradient can also be used by bacteria to eliminate antibiotics, which seems to be a factor involved in the prevalence of this enzyme in multidrug resistant microorganisms [25]. Na^+^-NQR consists of six subunits (A-F) and five confirmed cofactors [30–32], although a sixth cofactor has recently been proposed [33]. Subunits NqrB and NqrC, each contain a covalently-bound FMN molecule, linked via the unique phosphoester bond described above [5,15]. These two cofactors are involved in the internal electron transfer reactions of Na^+^-NQR, and participate in critical steps of the sodium pumping mechanism [14,15,34–36].

Bertsova *et al*. reported that the alternative pyrimidine biosynthesis protein–ApbE- (also known as Ftp, flavin-trafficking protein) plays an essential role in the assembly of Na^+^-NQR [17,37]. The ApbE gene is found directly downstream of the Na^+^-NQR operon, and it has been reported to be responsible for the incorporation of the FMN cofactors to subunits NqrB and NqrC, which was previously proposed to be autocatalytic [5,17]. In this work, *V*. *cholerae* ApbE was cloned, purified and characterized using one of the physiologic substrates of the reaction, NqrC. The data obtained reveals key aspects to understand ApbE activity *in vivo*, such as a high affinity for FAD, and a high selectivity for divalent cations. Moreover, novel regulatory mechanisms for the family are described, including the activation by ADP and K^+^, which indicate that the infection of the intestinal epithelium by *V*. *cholerae* upregulates ApbE activity and promotes the assembly of Na^+^-NQR. Finally, the analysis of the pH dependency of the activity allowed us to identify His257 as an important residue in the catalytic mechanism of ApbE. The data found in this report sheds new light into the regulation of the entire family and opens new avenues for research to understand the function of ApbE in bacterial cell physiology.

## Results

### Physical properties

**Fig 1:**
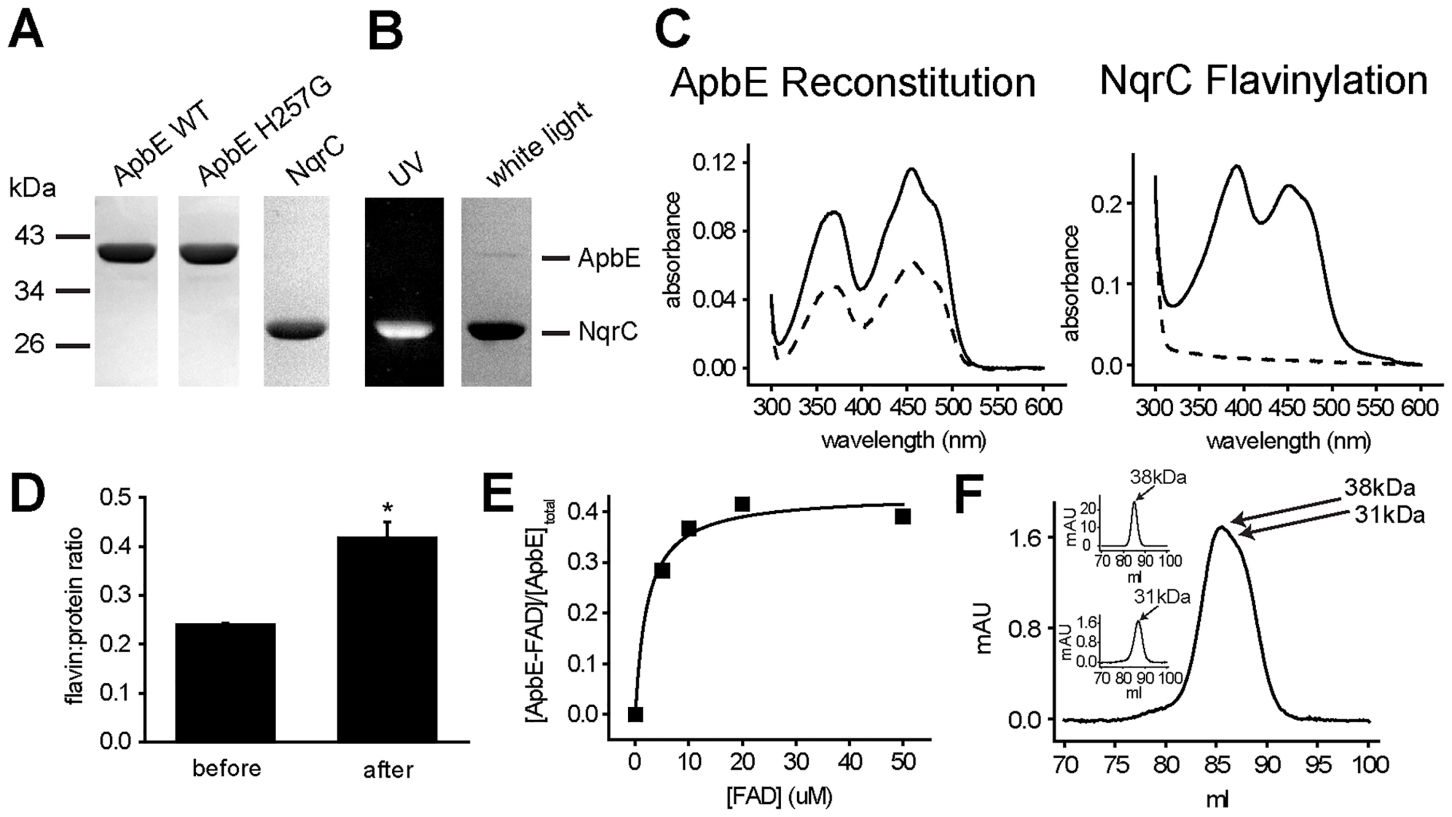
Physical properties of *V*. *cholerae* ApbE and NqrC. A) SDS-PAGE of purified WT ApbE, ApbE mutant H257G and NqrC. B) SDS PAGE of NqrC flavinylated *in vitro*. Left: unstained gel exposed to UV light; Right: Coomassie-Blue stained gel. C) Left panel, UV-*vis* spectra of ApbE before (dashed line) and after (solid line) reconstitution with FAD; right panel, UV-*vis* spectra of NqrC before (dashed line) and after (solid line) *in vitro* flavinylation. D) FAD:protein ratio of ApbE before and after reconstitution with FAD. *P < 0.05 (n = 3). E) Representative equilibrium dialysis experiment, measuring the binding of FAD to purified ApbE. F) Size exclusion chromatogram of purified ApbE (inset upper panel), NqrC (inset lower panel), and a mixture of ApbE and NqrC at a molar ratio of 1:1.

*V*. *cholerae* ApbE was cloned, overexpressed in *E*. *coli* and purified to homogeneity (purity >95%), using affinity (Ni-NTA) and ion exchange (DEAE) chromatographic steps (Fig 1A). The recombinant protein does not contain the leader sequence (first 17 amino acids), and it is obtained as a cytosolic protein, although it is naturally localized in the periplasmic space [17,38–40]. Size exclusion chromatography data indicate that ApbE is obtained as a monomer, with the expected molecular weight of 37 kDa (Fig 1F inset, upper panel). Previous reports indicate that ApbE’s from *Pseudomonas stutzeri* [19], *Treponema pallidum* [37] and *Thermotoga maritima* [41] are also isolated as monomers, while the enzymes from Enterobacteria, such as *E*. *coli* [37] and *Salmonella enterica* [39] are found as dimers.

Previous reports showed that *V*. *cholerae* NqrC can be heterologously expressed in *E*. *coli* cells as an apoprotein (lacking the FMN cofactor) [16], and thus can be used as a substrate in our enzymatic assays. The transmembrane segment of *V*. *cholerae* NqrC [17] was eliminated during the cloning procedure, and the protein was purified as a soluble cytosolic protein. After two chromatographic steps, a purity >95% was achieved (Fig 1A). NqrC was also obtained as a monomer, determined by size exclusion chromatography, with the expected molecular weight of 31–27 kDa (Fig 1F inset, lower panel).

To explore if these two proteins can interact and form a stable complex, size exclusion chromatography was performed to analyze a mixture of ApbE and NqrC at a molar ratio of 1:1. The data indicate that the complex is not formed under these conditions, since no significant peaks were observed before the two main protein peaks appeared (Fig 1F).

### Flavin binding assays

Purified ApbE has a distinctive bright yellow color, indicating the presence of tightly-bound flavin. The UV-*vis* spectrum of the purified sample shows the typical flavin absorption peaks at 360 and 450 nm (Fig 1C left panel dashed line), corresponding to a FAD: protein ratio of 0.24±0.01 (Fig 1D). The relatively low content of flavin compared to other ApbE preparations [37,42] can be explained by the substantial purification procedure carried out to obtain a highly pure sample. In other members of the family FAD tends to dissociate slowly during protein purification [37,42]. To corroborate that ApbE is purified in its active flavin-binding form, the sample was incubated with a molar excess of FAD (50:1) for 1 h, and washed thoroughly by four rounds of dilution (1:20) and re-concentration. The reconstituted sample exhibited a FAD: protein ratio of 0.42±0.05 (Fig 1C left panel solid line and 1D), similar to the flavin content of *P*. *stutzeri* and *S*. *enterica* ApbEs (0.7–0.8) [19,39], indicating that the purified ApbE preparation is indeed fully functional. The fact that the enzyme can retain FAD after the washing procedure indicates that the affinity for this substrate is high and that the *OFF* rate for FAD dissociation might be quite low (minutes to hours range).

The dissociation constant for FAD (K_D_^FAD^) was measured through equilibrium dialysis, as reported before [39], following the FAD/ ApbE ratio varying the concentration of FAD (Fig 1E). Using this method, we measured that K_D_^FAD^ = 2.2±1 μM, a fairly low dissociation constant, indicating a high affinity for FAD. Although *V*. *cholerae* ApbE K_D_^FAD^ is the highest reported so far, the affinity for FAD is comparable to the enzymes from *P*. *stutzeri* (K_D_ = 0.4 μM) and *E*. *coli* (K_D_ = 0.7 μM) [19,37]. The binding assays were performed in the absence of divalent cations, due to the basal FAD hydrolysis reaction carried out by ApbE in the presence of Mg^2+^ [42]. Although, divalent cations were not included in these experiments, the crystal structure [37,39] shows that Mg^2+^ does not play a major role in stabilizing the interaction of the protein with FAD, and thus no major changes in the dissociation constant are expected by including this cation.

### pH dependence of ApbE

Previous studies have clarified different enzymatic properties of ApbE [37,42,43]. However, in these reports ApbE activity was measured indirectly, following the hydrolysis of FAD [37,42,43], rather than the physiologic reaction in which the FMN cofactor is incorporated into the protein substrate. In this study, the flavin transfer activity to NqrC was measured directly, following the fluorescence under UV light of the flavinylated NqrC protein, separated by SDS-PAGE (Fig 1B). The UV-*vis* spectrum of *in vitro* flavinylated NqrC (Fig 1C right panel solid line) is similar compared to the spectrum of holo-NqrC produced *in vivo*, as reported before, showing a characteristic shit in the flavin absorption peaks, from 360 to 390 nm [17,43]. These experiments offer a better understanding of ApbE’s physiologic reaction and provide reliable data to characterize the mechanism and regulation of this enzyme.

**Fig 2:**
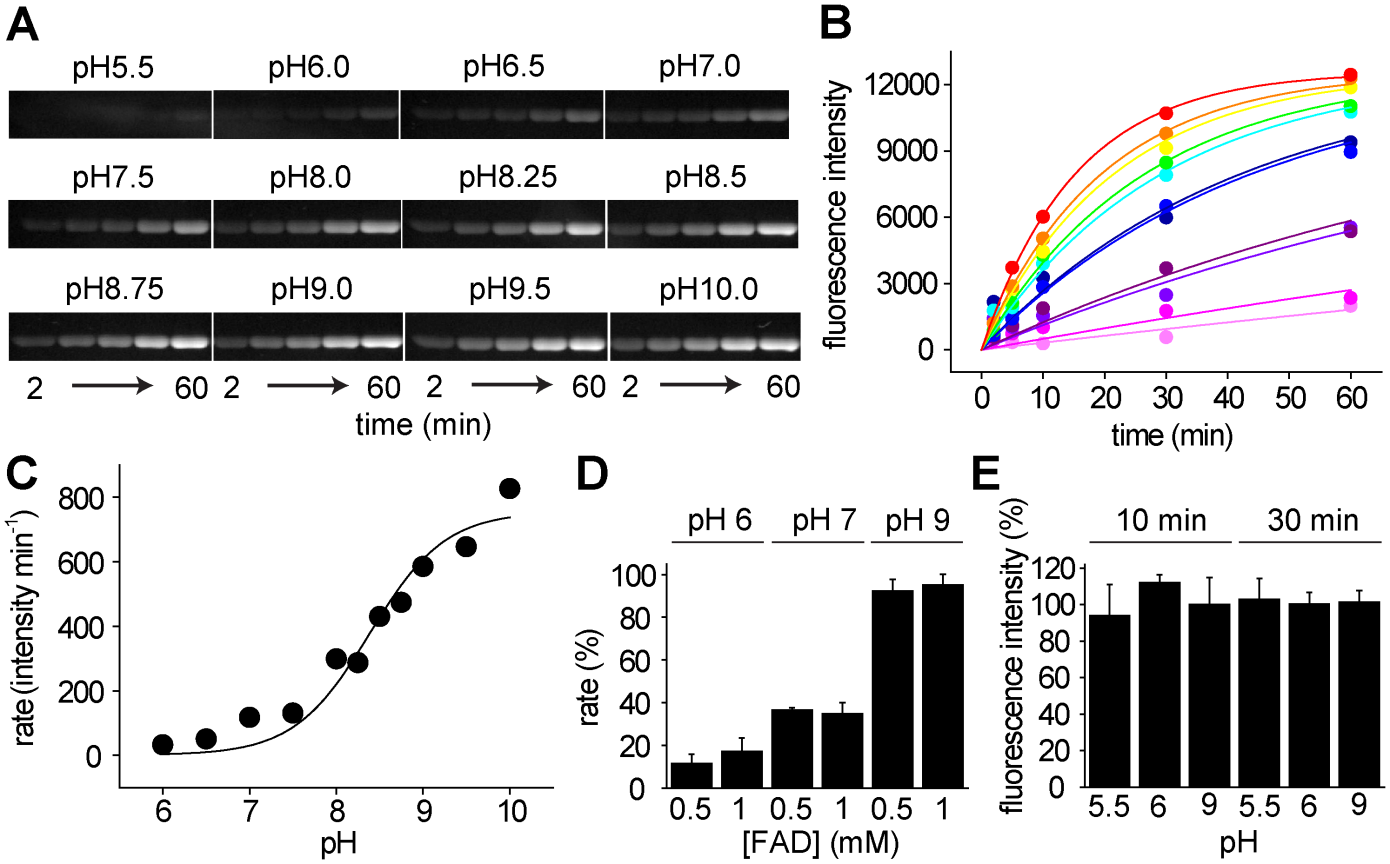
pH dependence of *V*. *cholerae* ApbE. A) Flavinylated NqrC ran through SDS-PAGE at different time points under different pH conditions. B) Kinetics of NqrC flavinilation at different pH values: pH 6.0 (Light magenta), 6.5 (magenta), 7.0 (violet), 7.5 (purple), 8.0 (dark blue), 8.25 (blue), 8.5 (cyan), 8.75 (green), pH 9.0 (yellow), 9.5 (orange) and 10.0 (red). C) Plot of the activity vs pH. Data were fitted using Eq 1. D) Effects of 0.5 mM and 1mM FAD on the reaction rate obtained at pH 6, 7, and 9 (n = 3). E) Effects of pH on ApbE stability. ApbE samples were preincubated for 10 and 30 min at pH 5.5, 6.0 and 9.0. The samples were diluted (1:20) in pH 9.0 buffer (Tris 100 mM) and the flavin transfer activity was measured in the same buffer (n = 3).

To understand the enzymatic and regulatory mechanisms of ApbE, a characterization of the optimum pH for activity was conducted. The experiments were performed in buffers of different pH values (5.5–10.0), using the reported substrates (1 mM FAD, 5 mM MgCl_2_, 10 μM NqrC) [17,19,42,43]. It should be pointed out that at pH 6, 7 and 9 the activity obtained with 0.5 and 1 mM FAD was nearly identical (Fig 2D). Thus, the concentration of FAD used in these assays can be considered saturating over most of the pH range tested.

The reactions were run for 1 h, and samples were collected at different time points and proteins were separated in SDS-PAGE (Fig 2A). The flavin transfer rates obtained at each pH (Fig 2B) were fitted to Eq 1 (Fig 2C), which describes the effect of pH on activity, providing information of the dependency of the catalytic process on the protonation state of the enzyme. As can be observed in Fig 2C, the activity *vs* pH plot shows only the ascending limb, with a pK_E1_ of 8.4, instead of the bell-shaped curve observed for other enzymes [44].

To corroborate that the pH dependency obtained is not the result of enzymatic inactivation at different pH values, ApbE was preincubated at pH 5.5, 6.0, and 9.0, for 10 and 30 min at room temperature. The samples were diluted (1:20) in pH 9.0 buffer and the activities were measured in this buffer. As shown in Fig 2E, no significant differences were observed after the treatment, indicating that ApbE is relatively stable in these conditions.

**Fig 3:**
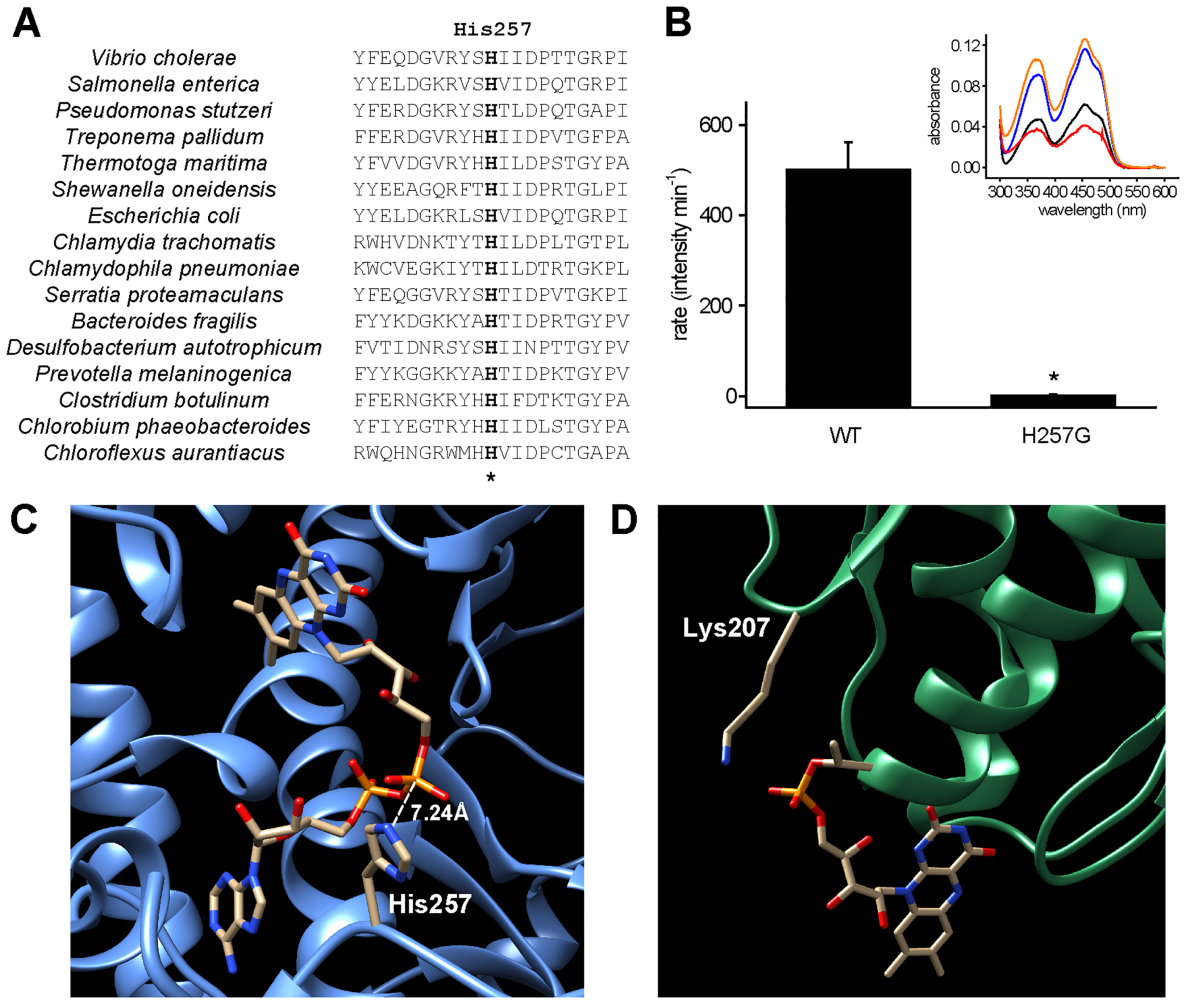
Role of ApbE-His257 and NqrC-Lys207. A) Partial amino acid sequence alignment of ApbE showing conserved His257 (marked with an asterisk). Amino acid sequences of ApbE were obtained from NCBI, and ClustalX was used to perform the alignment. The amino acid sequences around the essential conserved residues are shown. NCBI accession numbers for each sequence are: CSA56510.1 (*V*. *cholerae*), GAR62904.1 (*S*. *enterica*), KJH80864.1 (*P*. *stutzeri*), WP_014342584.1 (*T*. *pallidum*), AKE27441.1 (*T*. *maritima*), NP_716735.1 (*S*. *oneidensis*), CEE09930.1 (*E*. *coli*), CRH54071.1 (*C*. *trachomatis*), AAP98278.1 (*Chlamydophila pneumoniae*), WP_012005402.1 (*Serratia proteamaculans*), OCR36801.1 (*Bacteroides fragilis*), WP_015905056.1 (*Desulfobacterium autotrophicum*), WP_036864434.1 (Prevotella melaninogenica), KOR50543.1 (*Clostridium botulinum*), WP_012474569.1 (*Chlorobium phaeobacteroides*), YP_001637095.1 (*Chloroflexus aurantiacus*). Sequence numbering is based on *V*. *cholera*e ApbE. B) Flavin transfer activity of wild-type ApbE and H257G mutant. *P < 0.005 (n = 3). Inset, flavin absorption spectra of ApbE WT and H257G. Black, WT before reconstitution; blue, WT after reconstitution; red, H257G before reconstitution; orange, H257G after reconstitution. C) Crystal structure of *S*. *enterica* ApbE (PDB ID: 3PND) showing the conserved His257 at a distance of 7.2 Å to the β phosphate group of FAD. D) Crystal structure of *V*. *cholerae* Na^+^-NQR (PDB ID: 4P6V) showing the position of conserved Lys207 and the FMN acceptor Thr225.

Another explanation is that the pH dependence is the result of the protonation state of residues that directly participate in the transfer reaction. Therefore, this analysis is not only important to characterize the optimal pH of the reaction, but it also helps in the identification of possible catalytic residue(s) in the flavin transfer process, with a pKa close to pK_E1_. Analysis of ApbE sequence alignment was carried out to identify conserved residues with these properties. The results show the presence of a conserved histidine residue in position 257 (Fig 3A). The fact the nominal pKa (6.0) of Histidine residues is close to the pK_E1_ and that His257 is adjacent to FAD in the crystal structure (Fig 3C) strongly supports a major role in the catalytic mechanism. The mutant in which this residue is replaced by glycine (H257G) was purified and characterized (Fig 1A). Remarkably, the mutant shows a nearly total lack of flavin transfer activity (Fig 3B). However, after reconstitution, the H257G shows a FAD: protein ratio (0.48±0.02) nearly identical to the wild-type (WT) (Fig 3B inset, blue and orange lines), indicating that His257 is indeed essential for catalysis, but not for substrate binding. Other than His257, the conserved Lys207 in NqrC can also account for the pH dependency and has been found to play a critical role in flavin transfer from mutagenesis and crystallographic data (Fig 3D) [42]. However, this residue has a relatively high and inflexible pKa that makes it unlikely to fit in the pH profile obtained in this study (see Discussion).

### Substrate specificity: Divalent and monovalent cations

**Fig 4:**
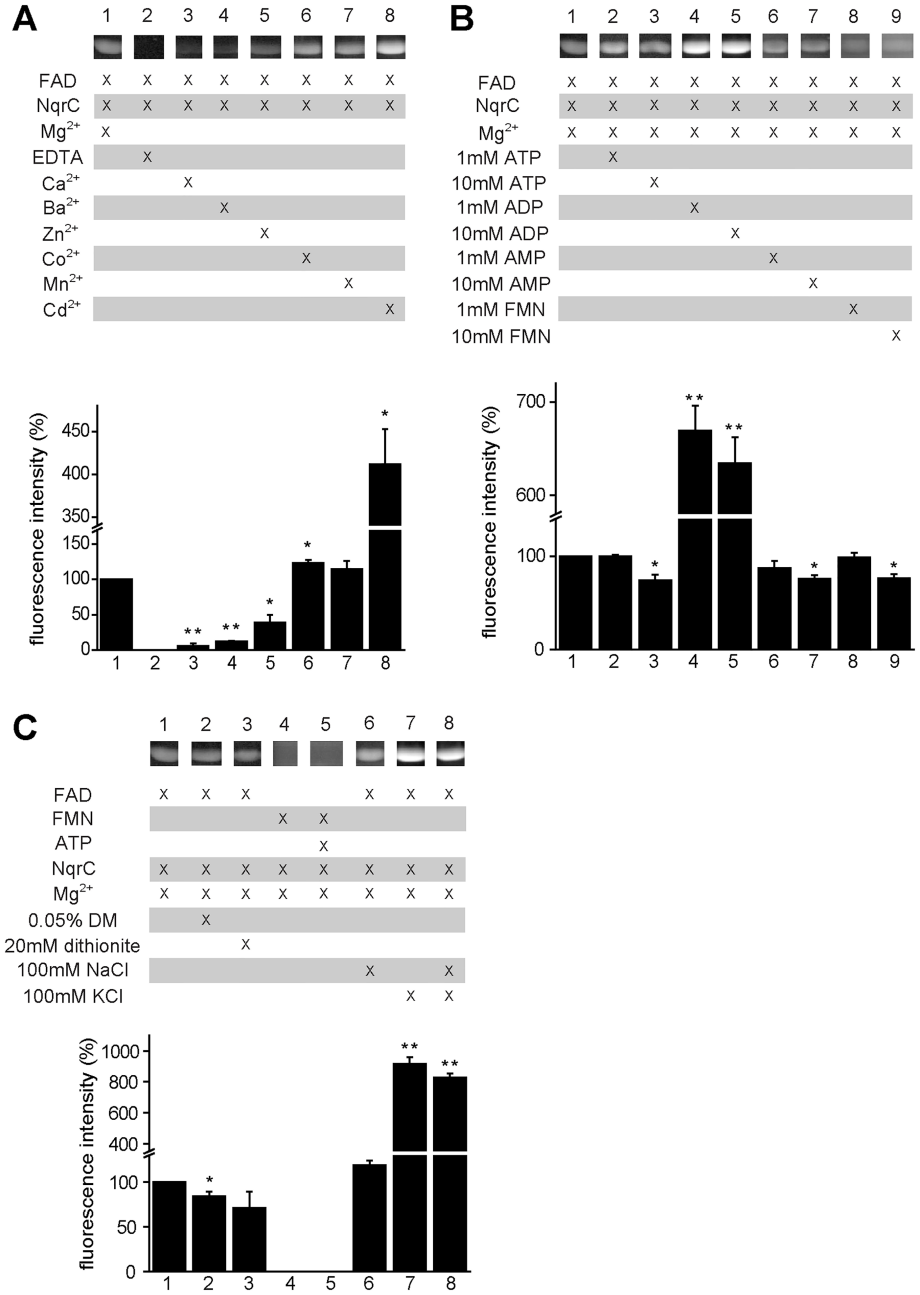
Substrate specificity of *V*. *cholerae* ApbE. Reactions were carried out as described in “MATERIALS AND METHODS” at pH 9.0. The reaction under physiologic conditions (10 μM NqrC, 1 mM FAD, 5 mM MgCl_2_) was used as control. Samples were collected after 10 min and mixed with 5X gel loading buffer. Samples were run in SDS-PAGE, which was later exposed to UV light. Student’s t–tests were used to determine statistical significance between control and experimental samples. *P < 0.05, **P < 0.005 (n = 3). Brightness and contrast of the images were linearly adjusted using Adobe Photoshop for better visualization. Original (unaltered) captured image files (not shown) were used for data analysis. A) ApbE activity was tested using EDTA or different cations. B) Effects of ATP, ADP, AMP and FMN on flavin transfer. C) Effects of 0.05% n-dodecyl-β-D-maltopyranoside (DM), 20 mM dithionite, FMN and monovalent cations on flavin transfer.

ApbE substrate specificity was characterized testing the effects on flavin transfer of analogs of the physiologic substrates reported previously: Mg^2+^, FAD and NqrC [17,19,42,43]. These experiments were done at pH 9.0, close to the optimum pH of the enzyme. Our data corroborates previous studies that show that divalent cations are essential for ApbE activity, and their removal by EDTA abolishes the activity [17,19,42,43] (Fig 4A, lane 2). Interestingly, the results indicate that ApbE is also able to use other divalent cations, such as Mn^2+^ and Co^2+^, obtaining a similar activity compared to Mg^2+^ (Fig 4A, lane 6 and 7). Moreover, the addition of Cd^2+^ significantly increased ApbE activity, by 4-fold (Fig 4A, lane 8). On the other hand, the activity obtained with Ca^2+^, which can replace Mg^2+^ in other nucleotide binding proteins [45], Ba^2+^, and Zn^2+^ was reduced (10–40%) (Fig 4A, lane 3–5). These results suggest that the coordination sphere largely determines the specificity of the enzyme for the divalent cation.

*V*. *cholerae* colonizes the upper and middle section of the small intestine [46,47], where it is constantly in the presence of Na^+^ and K^+^, both of which increase upon Cholera diarrhea onset [48–50]. To test if monovalent cations can regulate ApbE, the activity was tested in the presence of KCl and NaCl (100 mM). In contrast with divalent cations, monovalent cations are not essential for the activity. While the addition of sodium did not have a noticeable effect on activity (Fig 4C, lane 6), potassium produced a nearly 10-fold activation (Fig 4C, lane 7), which was not affected by the presence of NaCl (Fig 4C, lane 8), indicating that ApbE contains a highly specific K^+^ activatory site.

### Substrate specificity: Flavins and Nucleotides

The ability of ApbE to use other flavins, besides FAD, was also explored. Our results show that FMN is unable to be incorporated on its own to NqrC, even in the presence of ATP (Fig 4C, lane 4 and 5). Thus, the presence or hydrolysis of ATP’s high-energy bond, which is similar to the one found in FAD, is not involved *per se* in the flavin transfer reaction. Moreover, to investigate if ApbE has a preference for a specific redox state of FAD, 20 mM sodium hydrosulfite (a strong reductant) was added to the reaction. The concentration of hydrosulfite (freshly prepared as 1M solution and dissolved in Tris 1 M pH 8, to avoid drastic changes in pH) was enough to eliminate all oxygen dissolved in the sample buffer and to maintain FAD reduced for up to 8 h. The reduction of FAD did not produce significant changes in the flavin transfer activity (Fig 4C, lane 3), indicating that ApbE activity is independent of the redox state of the flavin.

Previous studies showed that molecules sharing common moieties with FAD, *i*.*e*. phosphorylated adenosine, such as ATP, ADP and AMP, act as inhibitors of the FAD hydrolysis reaction [37,42,43]. However, in our analysis the addition of ADP did not inhibit the transfer reaction, but significantly activated it, by 6-fold (Fig 3B, lane 4 and 5). On the other hand, 1mM of ATP, AMP or FMN had no effect on the activity (Fig 4B, lane 2, 6 and 8), but a higher concentration (10 mM), produced a slight inhibition (Fig 4B, lane 3, 7 and 9).

### Substrate specificity: Membrane environment

To simulate the membrane environment, 0.05% n-dodecyl-β-D-maltopyranoside, a mild detergent that preserves the quaternary structure of membrane proteins [51], was added to the reaction buffer, to assess if NqrC or ApbE require the hydrophobic environment (or the hydrophobic interface) for its proper function. A small (20%) inhibitory effect of the detergent was observed (Fig 4C, lane 2), indicating that ApbE may be able to operate entirely in the periplasmic space.

### Kinetic characterization of ApbE

**Fig 5:**
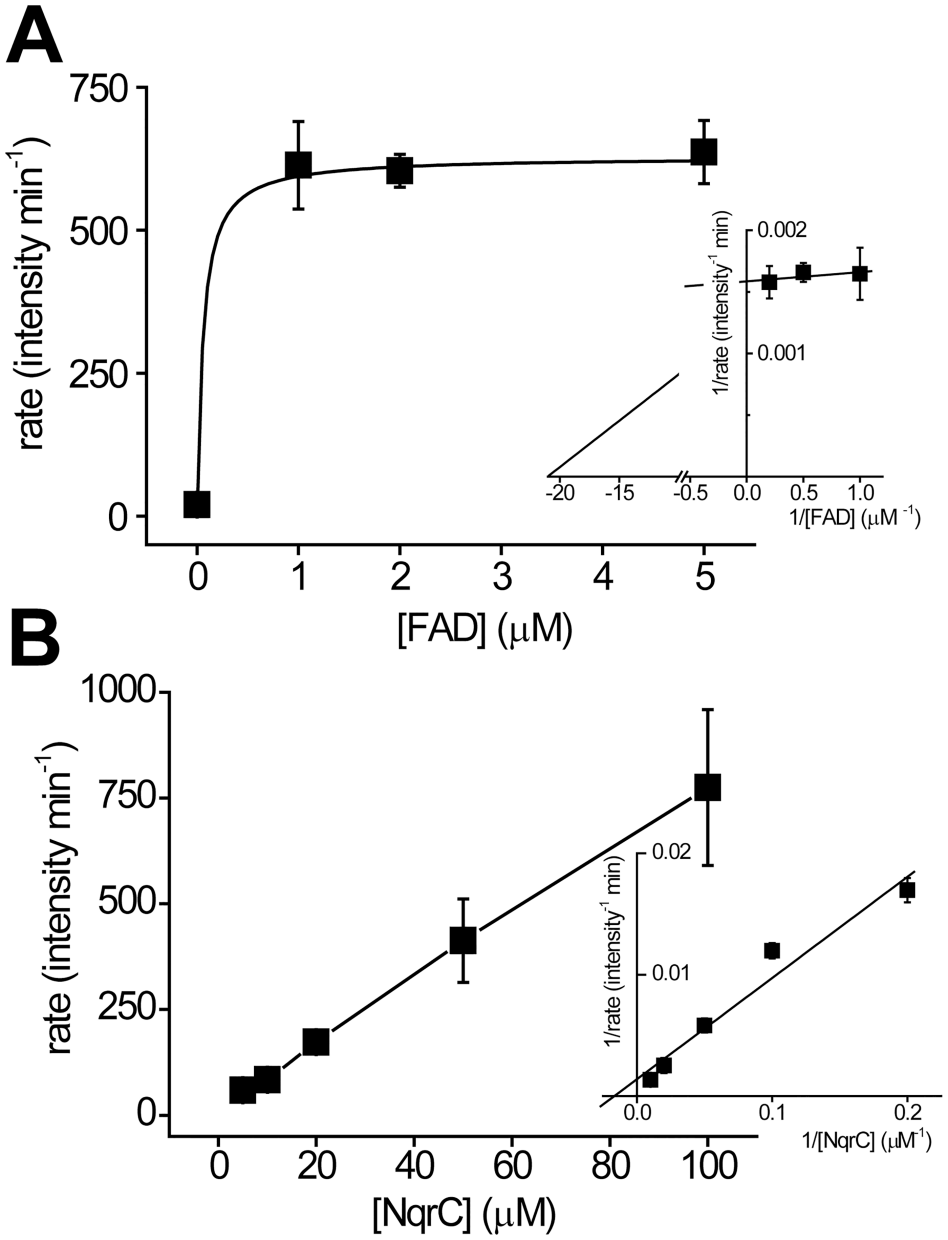
Substrate titration of ApbE. A and B) FAD and NqrC titrations on the steady state activity of ApbE, respectively. The titrations were performed by varying the concentration of one substrate at a fixed concentration of the other (FAD 1 mM, NqrC 100 μM). Inset shows the double reciprocal plots. The kinetic parameters were obtained by fitting the data to the Michaelis-Menten equation.

To have a better understanding of the mechanism of ApbE and its affinity for the substrates, a preliminary characterization of the steady state kinetics was carried out. The activity was measured at a high or nearly saturating concentration of FAD (1 mM) or NqrC (100 μM), while varying the concentration of the co-substrate. Our results show that ApbE activity is highly sensitive to FAD, and is saturated at 1 μM (Fig 5A). Although the method used in this work is robust, reliable data could not be obtained at low FAD concentrations, thus a rough estimation of the *Km* for FAD indicates that it falls in the sub μM range (Table 1).

**Table 1:** Kinetic parameters of *V*. *cholerae* ApbE.

Parameter	Value
*Km*_*FAD*_ (μM)	0.06 ± 0.25
*Km*_*NqrC*_ (μM)	> 100

On the other hand, the titration of the activity with NqrC can be described as a Michaelis-Menten behavior with a *Km* above 100 μM, showing a linear behavior even under fairly high substrate concentrations (Fig 5B and Table 1). These results show that ApbE has significantly different affinities to the substrates, which may provide insight into the physiologic working conditions of ApbE.

## Discussion

In most cases covalently-bound flavins are attached through a defined and well-characterized set of covalent bonds to the isoalloxazine ring, which include: 8α-*N*^3^-histidyl–FAD/FMN, 8α-*N*^1^-histidyl–FAD/FMN, 8α-*O*-tyrosyl–FAD, 8α-*S*-cysteinyl–FAD, 6-*S*-cysteinyl–FMN, and 8α-*N*^1^-histidyl-6-*S*-cysteinyl–FAD/FMN [5]. The covalent interactions increase protein and cofactor stability, but also modify the redox properties of the cofactor, which may favor a certain direction of the reaction [5,52,53]. The six-subunit respiratory complex Na^+^-NQR contains two covalently-attached FMN molecules, linked through a phosphoester bond between the phosphate group of FMN and conserved threonine residue in the sequence S(T)GAT [14–19]. The incorporation of the flavin cofactors to these proteins was considered an autocatalytic process [5,17]. However, Bertsova *et al*. described that the ApbE family is involved in the addition of covalent cofactors to the Na^+^-NQR complex [17]. In this work, we performed a detailed characterization of the kinetic properties of *V*. *cholerae* ApbE, to study its reaction and regulatory mechanisms.

### Divalent and monovalent cation specificity

Divalent cations play an essential role in the activity of nucleotide binding enzymes. For many enzymes the complex of the divalent cation with the nucleotide, *e*.*g*. Mg-ATP, is the true substrate of the reaction. The divalent cation stabilizes the phosphate groups and allows a proper conformation that in turn allows the nucleophilic attack required for phosphoryl transfer or bond hydrolysis [54,55]. Indeed, the crystal structure of *T*. *pallidum* ApbE reveals the presence of one Mg^2+^ ion coordinated with the pyrophosphate group of FAD, and a second Mg^2+^ interacting with the β phosphate group of FAD, as well as with Asp284, Ala162, Thr288, and a water molecule [37]. As reported previously, and corroborated by our data, divalent cations are essential for ApbE, and the addition of EDTA abolishes the activity [17,19,42,43]. Our results show that Mn^2+^ and Co^2+^ act as alternative substrates to replace Mg^2+^, due to their similar chemical properties, including a similar coordination pattern with a coordination number of six [56,57]. Intriguingly, the replacement of Mg^2+^ with Cd^2+^ led to a significant increase in the activity. The increase is too pronounced to simply indicate a higher rate of FMN transfer, and implies that the heavy metal may upregulate ApbE activity, triggering the production of Na^+^-NQR as a means of detoxification against heavy metals. On the other hand, Ca^2+^, Ba^2+^, and Zn^2+^ were unable to fully activate ApbE. Ca^2+^ and Ba^2+^ are poor substitutes for Mg^2+^ due to their large ionic radii [56,58]. Zn^2+^ coordination sphere commonly involves four ligands, while penta and hexacoordinates are less abundant [59]. Thus, Zn^2+^ might not be able to be bound to the Mg^2+^ sites in ApbE.

In contrast with *V*. *cholerae*, *P*. *stutzeri* ApbE is able to use Ca^2+^ as a substrate with an efficiency as high as Mg^2+^ [19]. Sequence alignment shows that the Mg^2+^ binding site of *P*. *stutzeri* ApbE is composed of Ser183, Asp297, and Thr301, two of which are completely conserved in various species, including *V*. *cholerae* [19]. However, in *V*. *cholerae* ApbE, Ser183 is replaced by a threonine residue (Thr171), leaving less space for divalent cation binding, which might explain why larger cations such as Ca^2+^ can substitute for Mg^2+^ in *P*. *stutzeri* ApbE, but not in *V*. *cholerae* ApbE. The results show that the ionic radius, as well as the coordination sphere of the divalent cation, play essential roles in ApbE substrate binding and catalysis.

The effects of monovalent ions were also investigated. Our results show that the enzyme is active in the absence of monovalent cations and that Na^+^ produces no significant effect. However, K^+^ triggers a 10-fold increase in ApbE activity. The K^+^ sensitive activation was not reversed by adding Na^+^ (100 mM) to the reaction buffer, suggesting that ApbE contains highly specific potassium regulatory site, which has not been identified in the crystal structure.

### Role of the membrane

ApbE activity was tested in membrane-like environment, using 0.05% DM as a detergent. This test is essential to understand ApbE function, since both protein substrates, NqrB and NqrC, are transmembrane proteins, and the cofactor binding sites are not readily available to the aqueous environment [33]. The addition of the detergent does not modify to a biologically significant extent the activity, and indicates that the role of ApbE is carried out entirely in the periplasmic space, with no associations with the membrane.

### Flavin specificity and regulation by adenine Nucleotides

To understand the role of the flavin donor, FMN was used directly as the substrate. The results show that ApbE was not able to incorporate FMN to NqrC, even in the presence of ATP, as a source of energy, indicating that ApbE lowers the activation energy of the flavin transfer process through hydrolysis of the FAD phosphoester bond, but the hydrolysis itself is not sufficient to energize flavin transfer. On the other hand, our data shows that although ApbE uses specifically FAD for flavin transfer, it does not have a preference for the reduced or oxidized form.

Potential inhibitors such as ATP, ADP, AMP, and FMN were assessed due to the fact that they share common moieties with FAD. However, no significant inhibitory effects of these molecules were observed, indicating that the high specificity of ApbE to FAD is based on both the isoalloxazine and the adenosine moieties. Remarkably, instead of inhibiting ApbE activity, ADP behaves as an activator, which might contribute to the pathogenicity of the bacteria (see below).

### pH dependency and catalytic mechanism

The characterization of the pH dependency over the activity shows that the optimum pH for ApbE is approximately 10, indicating that the protein must be deprotonated in order to carry out the reaction. These studies also demonstrated that the catalytic residue of ApbE might have a pKa of around 8.4, close to the pKa’s of histidine, cysteine, tyrosine, and lysine. The analysis of the sequence of ApbE proteins from different bacterial species showed that His257 is absolutely conserved. Moreover, the crystallographic data shows that His257 is in close proximity to the pyrophosphate groups of the FAD molecule, *e*.*g*. the distance between the β phosphate group of FAD and the N^ε2^ of His257 is 7.2 Å in *S*. *enterica* ApbE and 3.8 Å in *T*. *pallidum* ApbE. The difference in the distance between two species may suggest that His257 is located in a flexible loop that might alternate between several states and help carry the FMN molecule to NqrC. To investigate the role of His257, the mutant H257G was constructed and strikingly, it completely lacks flavin transfer activity. Previous studies by Deka *et al*. have shown that the mutation of the conserved Lys207 in NqrC eliminates the flavin transfer activity of ApbE, and this lysine was proposed as the catalytic reside for the family. In the crystal structures of *V*. *cholerae* and *Shewanella oneidensis*, NqrC Lys207 is found pointing directly at the acceptor threonine residue Thr225, which led to the proposal that Lys207 could deprotonate Thr225, facilitating the nucleophilic attack to the phosphate of FAD. Thus, it is possible that Lys207 could be the catalytic residue and can also be responsible for the pH dependent activation of ApbE [42]. Interestingly, *P*. *stutzeri* ApbE seems to be adapted to an acidic environment, and has a pH profile that is significantly different compared to *V*. *cholerae*, with an optimum pH of 7 and a pK_E1_ of approximately 6.2 [19]. The shift in the pKa of this residue in *V*. *cholerae* could be due to the adaptations of this bacteria to the intestinal environment (see below). This pKa is almost identical to the nominal pKa of imidazole (6.0), suggesting that His257 could indeed be the residue that determines the pH profile. On the other hand, a shift of 2–4 units in the pKa of Lys, from 10.5 to 8.4 or 6.2, would be one of the biggest changes reported. Studies indicate that changes of this magnitude can occur in lysine residues deeply buried in the protein core [60], which is not the environment expected in the hydrophilic cavity that holds FAD. Therefore, His257 is likely to account for the pH dependence of ApbE. The central role played by His257 seems to modify the proposed reaction mechanism [42], suggesting that His257 could help the nucleophilic attack on the phosphoester bond of FAD by the deprotonated Thr225. Although further work is required to fully elucidate the catalytic mechanism of ApbE, the data found in this paper will be crucial to gain a full understanding of the role of new residues in the reaction mechanism.

### Kinetic characterization of ApbE

Titrations of the two substrates of ApbE, FAD and NqrC, were performed to understand its kinetic mechanism. Consistent with the relatively low *OFF* rate of FAD, the kinetic data thus suggests that ApbE has a high affinity for FAD (*Km*_*FAD*_ < 1 μM) and that the binding of NqrC may facilitate the formation of the enzyme-FAD complex. This high affinity might indicate that the limiting step in the assembly of the Na^+^-NQR complex could be the transport of FAD from the cytosol, and that once FAD reaches the periplasm, it is trapped by ApbE. On the other hand, NqrC titration behaves as a typical Michaelis-Menten system, with fairly high *Km* -above 100 μM, consistent with the low ability of ApbE to form a stable complex with NqrC (Fig 1F). Such high *Km* for NqrC may suggest that to ensure Na^+^-NQR assembly, ApbE operates at a high concentration of the protein substrates in the periplasmic space [61].

### Flavin transport from the cytosol

The two subunits of Na^+^-NQR, NqrB and NqrC, that are able to accept the flavin from ApbE are located in the periplasm [33]. Moreover, the ApbE gene contains a leader sequence that directs it to this compartment [38]. An open issue regarding the physiologic role of ApbE is the source of FAD required for its function, since FAD is synthesized in the cytosol. Previous reports showed that *S*. *oneidensis* is able to secrete FAD from the cytoplasm, sustaining the extracellular electron transfer chain involved in iron metabolism [62]. Recently, a protein encoded by the gene *bfe* (*b*acterial *f*lavin adenine dinucleotide *e*xporter) was identified as the first bacterial flavin transporter in *S*. *oneidensis* [63], which is also present in many other bacteria, including Vibrio species. Bfe belongs to the Na^+^-driven MATE (multidrug and toxic compound extrusion) protein family. The function of this family of transporters is energized by the Na^+^ gradient [63,64], which is produced by Na^+^-NQR, indicating a tight co-regulation of the enzymes involved in sodium transport. A second type of flavin transporter was recently identified in *E*. *coli* (YeeO), also belonging to MATE family [65,66]. It is plausible that any or both of these flavin transporters are involved in *V*. *cholerae* to maintain the flavin homeostasis and ApbE activity.

### Proposed regulatory mechanism of ApbE

**Fig 6:**
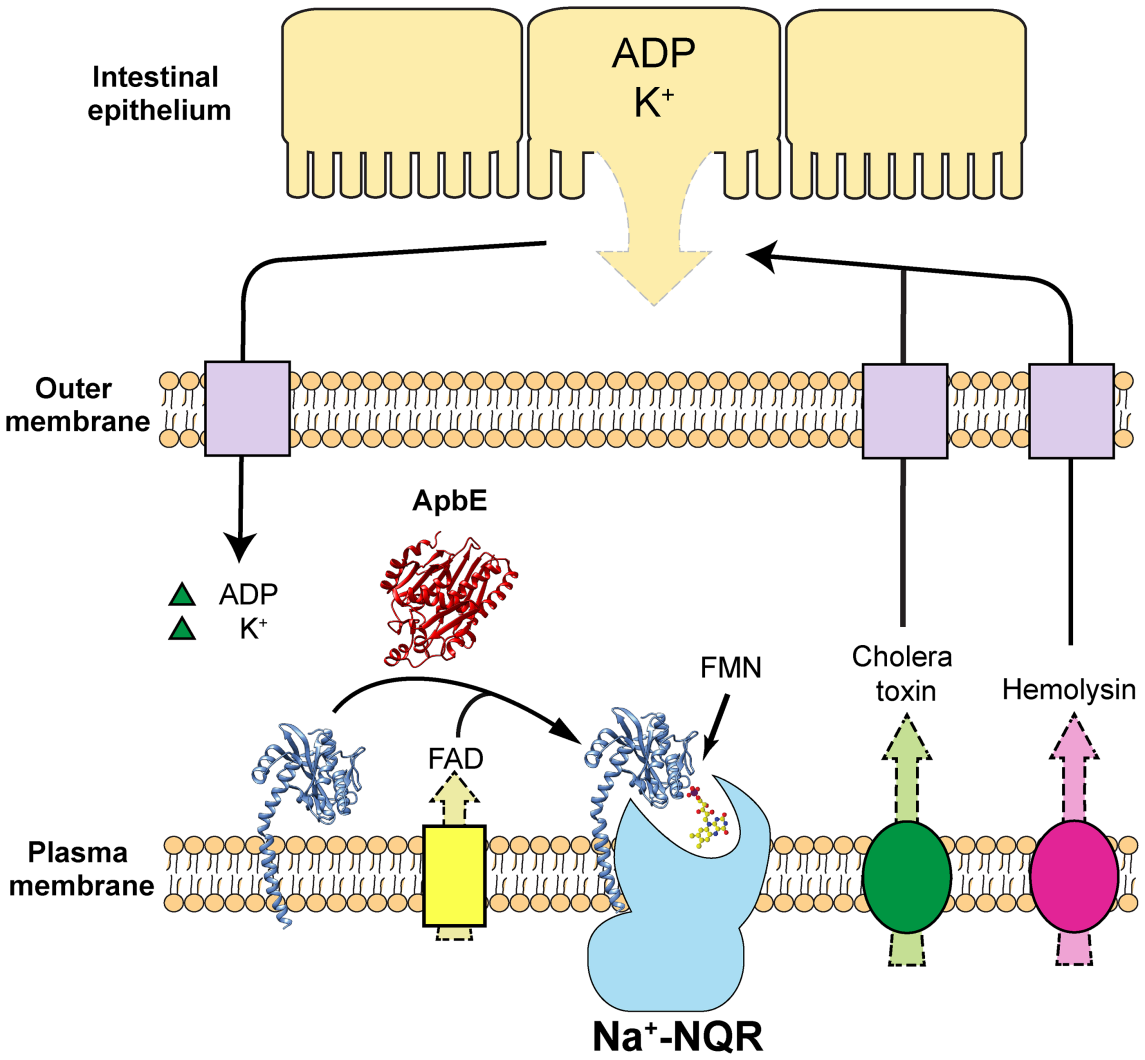
Proposed regulatory mechanism of ApbE. FAD transported from the cytosol binds ApbE which then post-translationally flavinylates NqrC, producing the holo-Na^+^-NQR. Hemolysin and cholera toxin produced by *V*. *cholerae* cause the lysis and leakage of the intestinal epithelial cells, leading to the release of ADP and K^+^, which serve as activators of ApbE.

Based on the results obtained, a regulatory mechanism can be proposed for ApbE that is related to the pathogenicity of *V*. *cholerae* (Fig 6). The colonization of *V*. *cholerae* of the small intestine, in particular the ileum where the pH is relatively basic (pH 7–8) [67], would lead to the stimulation of ApbE activity. FAD transported from the cytosol, or from the intestinal medium, would be used by ApbE as a substrate to post-translationally flavinylate apo-NqrC and aid in Na^+^-NQR assembly. The Na^+^-NQR complex thus is able to power the infection of *V*. *cholerae*, not only leading to the release of K^+^ via osmotic imbalance caused by the CFTR (cystic fibrosis transmembrane conductance regulator) Cl^-^ channel [48,50], but also the release of hemolysin [48]. The action of hemolysin can result in lysis of the intestinal epithelial cells [48], releasing ADP and more K^+^ [48,68]. These molecules and ions are then transported into the periplasmic space of *V*. *cholerae* and upregulate ApbE activity, further increasing the assembly of Na^+^-NQR and other respiratory enzymes, which in turn increase *V*. *cholerae* pathogenicity. Indeed, *V*. *cholerae* cells that lack Na^+^-NQR are almost entirely non-virulent [23,29]. The proposed regulatory mechanism is a positive feedback loop, in which the initial infection of *V*. *cholerae* leads to an enhanced infection through regulation of ApbE activity.

## Materials and methods

### Recombinant plasmid construction

In this study the flavin transfer reaction of the purified ApbE protein was characterized with one of its physiologic substrates, NqrC. These proteins were overexpressed as soluble cytosolic proteins, eliminating the leader sequence of ApbE [17,40,69] and the N-terminal transmembrane helix of NqrC [17], using PCR with the primers listed in Table 2. 6X His-tags were added to the C-terminus of both proteins, with a spacer of four glycines to prevent misfolding [70]. Amplified fragments were inserted into pBAD/HisB expression vector. The N-terminal *his-tag* sequence carried by this plasmid was eliminated through the addition of a stop codon. Moreover, a ribosome binding site was added upstream of the *apbe* and *nqrc* gene, as reported before [70]. *E*. *coli* DH5α cells were transformed with recombinant plasmids pBAD/HisB-ApbE and pBAD/HisB-NqrC. Sequences of the recombinant plasmids were verified by sequencing (Eurofins MWG operon). To express both proteins, the plasmids were introduced into *E*. *coli* BL21 cells.

**Table 2:** Primers used for gene amplification.

	Primer Sequence
ApbE WT forward primer	5’-TATGTCAGATCTTAGAGGAGGAATTAACCATGGAAAAGCCCGCAGAGCAGGTAC-3’
ApbE WT reverse	5’-AGCATCGAATTCCTAATGATGATGATGATGATGACCACCACCACCCTTACTTAAAAACGGTTTAAAGCTGCTTG-3’
NqrC WT forward primer	5’-ACGTACAGATCTTGAAGGAGGAATTAACCATGATCATTGTATCGGCAGCGGCTGTC-3’
NqrC WT reverse	5’-TATGTCGAATTCCTAATGATGATGATGATGATGACCACCACCACCGTTAAGACCTCCGTCACGAACTTTTGTC-3’
ApbE H257G sense	5’-GATGGCGTTCGTTACTCGGGTATCATCGATCCAACTAC-3’
ApbE H257G antisense	5’- GTAGTTGGATCGATGATACCCGAGTAACGAACGCCATC-3’

### Site directed mutagenesis

H257G mutation was performed using the Quikchange site-directed mutagenesis kit (Agilent technologies), using the primers listed in Table 2 and pBAD/HisB-ApbE as template [16]. The mutated sequence was verified by sequencing (Eurofins MWG operon).

### Protein expression

*E*. *coli* BL21 cells containing the expression plasmids of *V*. *cholerae* ApbE or NqrC were grown at 37°C in terrific broth media supplemented with 0.4% glycerol. Protein expression was induced at the middle of the log phase, adding 0.05% (w/v) arabinose. Cells were harvested by centrifugation at 3,100 x *g* for 20 min. Cell pellets were washed twice with washing buffer (300 mM NaCl, 1 mM MgCl_2_, 5 mM imidazole, 50 mM Na_2_HPO_4_, pH 8.0), and were resuspended in this buffer supplemented with 1 mM PMSF. Cells were then sonicated five times for 1 min with pulses at 50% duty cycle, using a Sonifier^®^ Cell Disruptor 350 (Branson). Cell debris was eliminated by two centrifugation steps at 4,700 x *g* for 30 min and 32,000 x *g* for 30 min. The supernatant was ultracentrifuged at 67,000 x *g* for 3 h, to eliminate membrane fractions. All centrifugation steps were carried out at 4°C. The supernatant was collected as membrane-free cell extracts, and was used for chromatography.

### Protein purification

Filtered membrane-free cell extracts were applied to Ni-NTA Sepharose 6 Fast Flow column (GE healthcare). The column was washed with buffer containing 300 mM NaCl, 20 mM imidazole, 50 mM Na_2_HPO_4_, pH 8.0. Proteins were eluted using a linear imidazole gradient (20–300 mM). Samples were concentrated with Amicon Ultra-15 centrifugal filter units (Merck Millipore) and stored at -80°C. Both proteins were further purified by diethylaminoethyl (DEAE) cation exchange chromatography. Samples were applied to DEAE Sepharose Fast Flow (GE Healthcare). The column was washed with buffer containing 50 mM Tris-HCl, 1 mM EDTA, 5% glycerol and a NaCl gradient (0–500 mM) was applied. Protein purity was higher than 95%, as shown by SDS-PAGE (Fig 1A). Samples were collected, concentrated and stored at -80°C until further use.

### Gel filtration

To determine the oligomeric states of ApbE and NqrC, size exclusion chromatography was performed with HiLoad 16/600 Superdex 200 pg column (GE healthcare). ApbE and NqrC samples were run through the column in buffer containing 150 mM NaCl, 10 mM HEPES, pH 8.0. To study the formation of complexes of ApbE and NqrC, the two proteins were mixed at a molar ratio of 1:1 and the mixture was then run through the column, as described above.

### ApbE activity measurements

ApbE catalyzes the flavin transfer to NqrC, using FAD as substrate, producing AMP. Flavinylation of NqrC can be followed in SDS gel exposed to UV light, due to the fluorescence of FMN [14–16], offering a relatively simple and reliable method to follow ApbE physiologic activity. Samples were collected at different times and the reaction was stopped by adding 5X SDS-PAGE loading buffer. Samples were run in SDS-PAGE, and the gel was exposed to UV light. Fluorescence intensity was analyzed the original (unaltered) gel pictures using ImageJ [71].

### Substrate specificity

To investigate the substrate specificity of ApbE, different combinations of analogs of NqrC, FAD, and Mg^2+^ were used as listed in Fig 4. The control reaction was set up with 0.3 μM ApbE, 10 μM NqrC, 1 mM FAD, and 5 mM Mg^2+^ in buffer containing 100 mM NaCl, 1 mM EDTA, 25 mM Tris, pH 9.0. The substrate analogs were added at the same concentration as the reported substrates. Moreover, in reaction conditions that contained 10 mM of ATP, ADP, AMP, or FMN, the concentration of MgCl_2_ was increased to 15 mM, since these molecules act as magnesium chelators. All reactions were run for 10 min, stopped and run through an SDS-PAGE fluorescent analysis, as described above. Control reactions were loaded in each gel, along with reactions under different conditions for normalization.

### ApbE pH-dependence assay

This assay was carried out at room temperature using 10 μM NqrC and 1mM FAD as substrates, in buffer containing 100 mM NaCl, 1 mM EDTA, 5 mM MgCl_2_, 25 mM MES, 25 mM HEPES, 25 mM Tris, at different pH values (5.5–10.0). Reactions were started by adding 0.3 μM of ApbE. Rates were obtained by plotting fluorescent intensities against time at a near saturating concentration for FAD, and were fitted to the following equation describing the behavior of the activity when the pH is varied:

$$v=\frac{Vmax*[S]}{Km*[1+\frac{pH}{{pK}_{E1}}]+[S]*[1+\frac{pH}{{pK}_{E1}}]}$$ (1)

where *v* is the reaction rate, *Vmax* is the maximum reaction rate, [S] is the concentration of NqrC used in this assay, *Km* is the Michealis constant of the enzyme and pK_E1_ is the negative logarithm of K_E1_, the dissociation constant of the protonated enzyme [44].

### Steady state kinetic measurements

To obtain the kinetic parameters of ApbE, titrations of the two substrates, NqrC and FAD, were performed using various concentrations of each substrate and a fixed concentration of co-substrate (100 μM NqrC or 1 mM FAD). The reaction buffer contained 100 mM NaCl, 1 mM EDTA, 5 mM MgCl_2_, 25 mM Tris, pH 9.0. ApbE (0.1 μM) was added to initiate the reaction, which was terminated at different time points. The rates were plotted against substrate concentrations and fitted to Michaelis-Menten equation to obtain the kinetic parameters.

### Flavin determination

UV-*vis* spectroscopy was used to determine the flavin content of the samples. ApbE sample was diluted in buffer containing 150 mM NaCl, 1 mM EDTA, 20 mM HEPES, pH 7.5. UV-*vis* spectra (300–700 nm) were obtained using Cary 8454 UV-*vis* diode array system (Agilent Technologies). The molar extinction coefficient of FAD (ε_FAD_) used for calculations was 12.5 mM^-1^∙cm^-1^ at 450 nm [72].

### Flavinylated NqrC spectrum

To determine if the *in vitro* flavinylated NqrC has the same spectral properties as the *in vivo* flavinylated NqrC reported before [17,43], the spectrum of holo-NqrC produced by ApbE *in vitro* was investigated. Reaction was set up in buffer containing 100 mM NaCl, 1 mM EDTA, 5 mM MgCl_2_, 25 mM MES, 25 mM HEPES, 25 mM Tris, pH 9.0. 0.1 μM ApbE and 50 μM NqrC were added to the buffer which was then subjected to UV-*vis* spectroscopy as control. Reaction was initiated by adding 1 mM FAD and incubated for 4 h. To remove free FAD, the sample was washed with the buffer described above (1:20 dilutions) three times and was then used to measure the spectrum.

### ApbE reconstitution

To examine if the purified ApbE retains the flavin binding capability, the sample was reconstituted with FAD. 20 μM ApbE and 1 mM FAD were incubated in reconstitution buffer (150 mM NaCl, 50 mM Tris, pH 8.0) at room temperature for 1 h. The sample was washed four times with reconstitution buffer to remove any free FAD. The sample was then subjected to UV-*vis* spectroscopy to detect tightly bound flavins at 360 nm and 450 nm.

### FAD binding assays

To investigate the flavin binding capability ApbE, equilibrium binding experiments were performed using Pierce^®^ 96-well Microdialysis Plate (Thermo scientific). Purified proteins were washed twice with reconstitution buffer. After the washing was completed, 50 μM of the protein samples were added to the microdialysis devices (100 μl). Dialysis was carried out in reconstitution buffer (1.5 ml) (supplemented with different concentrations of FAD) at 4°C with constant shaking for 14 h. The concentrations of FAD in the internal and external chambers were measured, which allowed us to calculate the K_D_ for FAD.

## Supporting information

S1 File Original data for “Kinetic characterization of *Vibrio cholerae* ApbE: Substrate specificity and regulatory mechanisms”. (XLSX)
